# Mentorship during transition period: a challenge for newly qualified midwives in Limpopo province of South Africa

**DOI:** 10.4314/ahs.v22i1.25

**Published:** 2022-03

**Authors:** Khathutshelo Simane-Netshisaulu, Maria Maputle, Lizzy Mutshinyalo Netshikweta, Hilda Shilubane

**Affiliations:** University of Venda, Advanced Nursing Science

**Keywords:** Experienced midwives, mentoring, mentor, newly qualified midwives

## Abstract

**Background:**

Mentorship is a process in which structured support is provided to new graduates of the profession to facilitate theireffective transitional journey to professional autonomy.

**Objectives:**

To explore and describe the mentoring process as experienced by newly qualified midwives and experienced midwives during thetransition period.

**Methods:**

Aqualitative approach was used. Five hospitals were selected from Limpopo province. The study was conducted in a maternity unit of each selected hospital. Population comprised of all newly qualified midwives as well as all experienced midwives working at institutions under study. Non-probability, purposive sampling method was used to select twenty-five newly qualified and twenty-five experienced midwives working in maternity wards of selected hospitals. In-depth face-to-face interviews were conducted for data collection.

**Results:**

Ineffective mentoring processes were reported, where only a few experienced midwives seemed ready to provide informal and unstructured support to graduates. Experienced midwives recognised their mentoring role however, felt they did not have sufficient knowledge and skills regarding mentoring process. Shortage of staff and increased workload were reported as challenges which negatively affected the mentoring process.

**Conclusion:**

Mentoring is an effective process for facilitation of graduates' transition process to become registered autonomous midwifery practitioners. However, they were not effectively mentored; consequently, negatively affecting their development to professional maturity.

## Introduction

Transition from being a midwifery student to becoming a registered midwifery practitioner is a stressful process which demands provision of structured support and guidance through mentorship to achieve competence; without which newly qualified midwives are at risk of developing anxiety resulting in reality shock^1^,^2^,^3^. Mentorship is described as a one-to-onerelationship between a more senior staff member and a junior member aiming at support and development of work-related skills^4^. When effectively done during thetransition period, mentoring promotes effective transition from being a student to becoming a registered autonomous midwifery practitioner; mainly concerned with confidence building based on a more personal relationship rather than assessment of competence^4^.

In a study conducted by Kensington, Campbell, Gray, Dixon, Tumilty, Pairman, Calvert and Lennox, mentors played an important role in helping midwifery graduates regarding setting of goals, debriefing and identification of learning areas^5^. Adhikari and Nsubuga as well as Kemp shared the same sentiments when they emphasised that, mentoring is of vital importance as it promotes production of quality nurses and midwives^6^,^7^. According to Zhang, Qian, Wu, Wen and Zhang, the use of trained mentors in the mentoring programs for graduates, have demonstrated promising success in facilitating the graduates' transition process^8^.

According to the Ugandan Nurses and Midwives Council (UNMC), mentoring should be provided by a mentor who is defined as a nurse or midwife who has completed an accredited mentorship training by the Council and is skilled, knowledgeable and with a positive attitude to guide students according to their learning needs^9^. In New Zealand, workshops aiming at empowerment of mentors regarding the mentoring role were conducted yearly; and not only did mentors attend, but even midwives who did not form part of the mentoring process showed interest. This played a vital role regarding improvement of the mentoring role^5^. The findings of the study conducted by Zhang, Qian, Wu, Wen and Zhang revealed that, for thementoring process to be effective and objective, collegial relationship between an experienced member and a neophyte should be positive^8^. The authors further emphasised that the mentor should be competent regarding the mentoring role8. Based on Mubeezi and Gidman'sfindings, participants reported compromised standard of mentoring in Uganda as they lacked equipment and had limited time due to their increased responsibilires.^10^

Based on literature from the Royal College of Midwives (RCM), lack of support of newly qualified midwives, contributes to attrition ranging from five to ten percent whereby graduates leave the profession during the first year of practice^11^. Adhikariand Nsubuga reported provision of compromised care to patients, which resulted from failure to provide effective mentoring process to newly qualified midwives^6^.

The South African National Department of Health (SANDoH) reported that the duration of training for a comprehensive nursing programme, especially in relation to midwifery, is perceived to be too accelerated and does not give student midwives requisite competencies; thus limiting their ability to function within the scope of practice prescribed for registered midwives^12^. It is for this reason that the South African legislation (RSA, Act Number 33 of 2005) prescribed that all newly qualified midwives who have undergone a comprehensive programme (SANC Regulation, R425 of 19 February 1985, as amended) be placed in public hospitals to complete one year of compulsory service^13^,^14^. During this placement, graduates are placed in maternity unit for six months, whereas the other six months are spread in medical, surgical, outpatients' department and casualty, paediatric, theatre and psychiatric units respectively. The aim of compulsory placement is that they be orientated, supervised and mentored in their new role before registration as professional nurses by SANC. This is supported by Simane-Netshisaulu, Maputle, Netshikweta and Shilubane, who reported that facilitation of effective transition of graduates is determined by effective implementation of mentoring programme^15^.

The researchers are lecturers responsible for midwifery students' learning in Limpopo province. During accompaniment of midwifery students at the hospitals, the researchers observed on several occasions that newly qualified midwives placed in maternity units of the hospitals where midwifery students were placed for clinical learningwere acting as the most seniors staff members in charge of a labour ward; taking full accountability for the patients as well as junior staff members including students. This is contrary to the aim of midwifery graduates' compulsory placement during transition period. Failure to provide effective mentoring process to newly qualified midwives result in provision of compromised care to patients^5^. It is in this light that the researchers conducted the study aiming to explore and describe the mentoring process as experienced by newly qualified midwives and experienced midwives during transition period.

## Methods

### Study Area

A qualitative approach, with anexploratory, descriptive and contextual design was used to explore and describe mentoring process during transition periodfor newly qualified midwives. One hospital was selected from each of the five districts of Limpopo province, and the study was therefore conducted in five maternity units from the five selected hospitals.

### Source and Study Population

Population comprised of two groups: (1). All newly qualified midwives who have undergone a comprehensive nurses' training programme based on R425 of 19 February 1985 as amended, and qualified as nurses (general, psychiatric and community) and midwives; as well as (2). All midwives practicing in hospitals selected for the study in Limpopo province.

### Inclusion and Exclusion Criteria

All newly qualified midwives working in maternity during their first year of clinical practice following graduation were included in the study, whereas those working in general wards were excluded. Those who have been practicing for more than a year were also excluded from the study.

Experienced midwives who have been working in thematernity unit for at least five years were included in the study. Experienced midwives whose work experience in maternity was less than five years as well as those who were not working in maternity during conduction of the study were excluded.

### Sample Size and Sampling Procedure

Two groups of participants were sampled using anon-probability, purposive sampling strategy, as follows: (1). Five newly qualified midwives working in amaternity unit during their first year of clinical practice following their graduation, as well as; (2). five experienced midwives who have been working in amaternity unit for at least five years.

The sample was made up of twenty-five newly qualified midwives and twenty-five experienced midwifery participants.

### Data Collection Procedure

Individual interviews were conducted as means of data collection; as the researchers aimed at obtaining in-depth information regarding mentoring process as experienced by newly qualified midwives and experienced midwives during transition period. A voice recorder was used to capture participants' responses after obtaining their permission. An open-ended question was posed as: “*What are your experiences regarding mentoring of newly qualified midwives during transition period*?” Probing was done to facilitate unrestricted expressions and responses from the participants. Observation of participants was done during the sessions. Field notes were documented during interviews and were given meaning through the researcher's reflections. Each interview session lasted for about an hour; and enabled the researcher to explore the mentoring process for newly qualified midwives. Interviews were conducted until data saturation was reached.

### Data Quality Control

Criteria for ensuring trustworthiness based on Lincoln and Guba's principles as described in De Vos, Strydom, FoucheandDelport were ensured^16^. Credibility was ensured by prolonged engagement which enabled researchers to establish rapport and build trust with participants. Researchers spent time listening to and observing participants during interviews; which were conducted until data saturation was reached. Recorded interviews were transcribed verbatim and nonverbal cues such as frowns and head shaking were written within brackets to ensure authenticity. By so doing, researchers ensured confirmability. An independent coder analysed raw data to develop themes. Final themes and subthemes were developed after an agreement was made by an independent coder and researchers. This was done to ensure dependability. Member checking was also done formally after data had been fully analysed. Thus, the preliminary findings of the research were discussed with the participants to validate the results. Transferability was ensured by thick descriptions of research methodology.

### Data Processing and Analysis

Qualitative data analysis was done using Tesch's open coding method including the following steps: careful reading of all the transcripts by the researcher to get a sense of whole; compilation of a list of similar topics; grouping of data according to themes and sub- themes and coding and categorization of field notes. Results were contextualised through literature control^17^.

## Results

The findings emerged from the interviews conducted with two sampling groups as follows: (1). Twenty-five newly qualified midwives placed in maternity units of selected hospitals.

These were graduates practicing during their first year following their completion of training. Five graduates were sampled from each hospital. Females constituted 94.8% whereas males composed 5.2% of the total sample. In terms of ethnic groups, 38.8% were Pedi, 39.1% Tsongas, 8.7% Swati and 13.4% Venda. Of all the participants, 70% have undergone their training at the universities whereas 30% undergone their training at the college of nursing. Newly qualified midwives shared their experiences regarding the mentoring process during the transition process. (2). Twenty-five experienced midwives who have been practicing in maternity units for at least five years.

Five experienced midwife participants were sampled from each hospital. All participants were females. In terms of ethnic groups, 22.5% were Pedi, 23,6% Tsongas, 12% Swati and 41,9% Venda. Data from participants were consolidated and linked to each other to form clusters; two themes and four sub-themes emerged as presented in Table 1.

**Table 1:** 

Themes	Sub-themes
1. Newly qualified midwives' experiences regarding mentoring	1.1. Mentorship: Does it exist? 1.2. Midwifery units: How conducive are they for learning?
2. Experienced midwives' views regarding mentoring role.	2.1. Mentors: How ready are they? 2.2. Shortage of staff and increased workload: How burdensome are are they for mentoring?

### Themes and Sub-themes

#### Newly qualified midwives' experiences regarding mentoring

Newly qualified midwives expressed dissatisfaction about the mentoring process, especially because the environment in the midwifery units was non-conducive for learning and support.

### Mentorship: Does it exist?

Serious concerns were raised by newly qualified midwives regarding lack of formal mentoring process, which negatively affected their transition process. Based on the findings, experienced midwives seemed not to be ready to take responsibility for their role as mentors.

According to participants, clinical placement during the first year of transition was meant for mentoring; but to their surprise, they were expected to work as if they were experienced. Participants expressed their frustration regarding lack of supervision and mentoring, especially during conduction of labour. They reported that experienced midwives do not allocate time for attending to them they just observe them as they pass. Graduates are referred to their colleagues for assistance if experiencing problems. This was reflected in the following participants' quotes:

“*Some experienced midwives observe you as they pass, if you are doing something right they just keep quiet. If what you are doing is wrong, some will just say ‘do this and this’. Some say ‘ask your colleagues to help you. This is bad because as a new graduate, I need somebody who will take me by my hand and show me the way as my mentor.” “Honestly speaking there is no supervision and mentoring. When a patient is in labour, I progress her and even deliver her alone without any supervision or assistance. It is really unfair*.” Newly qualified midwives appreciated a very few experienced midwives who demonstrated a certain degree of willingness to assist; though the help they offered was not formal and structured. The findings revealed that newly qualified midwives were not satisfied about the way support was offered to them during transition period as mentoring process was undermined.

### Midwifery units: How conducive are they for learning?

Graduates described midwifery units as being non-conducive for effective transition, as some of the questions they had regarding practice were not positively attended to, instead they were expected to practice as though they were experts in the midwifery field. The environment was considered unsupportive and therefore hindered graduates' growth during transition period.

Newly qualified midwives described the environment in maternity unit as nonconductive for learning, as their learning needs were notmet. When graduates asked questions, theywere told that there is no time to attend to their questions as the ward was busy. Graduates were also told that the ward environment was not meant for teaching but practice. ‘All you have to do is to work since you have passed your examinations.’

Participants needed somebody with experience to boost their confidence as they provided care to patients; especially during performance of difficult procedures such as resuscitation of a new-born baby. Learning through trial and error was the order of the day, which really put patients' lives at risk.

“*It is not that we were not taught during training....no, we were taught, and we know how to do these procedures. But you need somebody who is experienced to support you as you stand so that you gain confidence to perform procedures as an independent practitioner.” “The situation is not good at all; in some instances, you have to learn through trial and error. I was so scared of resuscitating a newborn baby, until one day in which I had to practice it all by myself. Fortunately, the baby cried whilst I was still struggling with the tubing. We were well prepared academically, but you need to have more time in scary areas such as resuscitation of a new-born baby*.” Failure to receive effective support in the midwifery units in which graduates were placed, did not only have a negative impact on their performance, but their confidence level too.

### Experienced midwives' views regarding mentoring role

Experienced midwives expressed positive attitudes towards their role as mentors, but raised concerns regarding their level of preparedness on the management of the responsibilities accompanying the mentoring role as well as shortage of staff.

### Mentors: How ready are they?

It was evident from the findings of the study that participants recognised their role inmentoring and supportinggraduates however, they felt they did not have sufficient knowledge and skills regarding the mentoring process.

Experienced midwives expressed a need to be empowered so that they could be up to date with the new developments related to provision of quality midwifery care. They also needed to be trained on how they canprovide mentoring process.

One participant said: “*My problem with mentoring of the graduates is that, things are always changing in the health system. Maybe you need some refresher courses so that you are up to date*.”

Another participant confirmed: “*I did my training a long time ago and a lot has changed since then. For me to be able to support them I need some training on how to do it. Otherwise, I don't think I can.” “Mentoring is important, and I was also mentored when I was a junior member in this profession. But, for you to do it effectively, you need some guidance on how to do it*.”

Participants expressed their willingness to take their mentoring role, however lack of readiness and preparedness in terms of knowledge and skills regarding the mentoring process, failed them.

### Shortage of staff and increased workload: How burdensome are theyfor mentoring

Experienced midwives raised issues of shortage of staff and increased workload as factors that hindered their ability to fulfil their mentoring role. The fact that they are being overworked, makes it difficult for them to effectively mentor newly qualified midwives even if they would want to.

Experienced midwives reported that they gave priority to provision of patients' care, as they are expected tocare for patients despite staff shortage. To experienced midwives, mentoring and supervision of graduates are time consuming, therefore result in compromised patients' care. The following participants' quotes serve as confirmation: “We are short staffed... you find that most of the time we are few and even if you want to mentor them, you can't because the ward is very busy. Therefore, you give priority to the patients.”Another participant said: “The workload is very high in such a way that there is no time for mentoring. You are on your feet most of the time, the little time you get you are so exhausted, you can't even think well.”

Shortage of staff and increased workload were reported as serious challenges that negatively impacted on experienced midwives' role as mentors.

## Discussion

The findings of the study revealed that mentoring of newly qualified midwives during their transition period was ineffective. Only a few experienced midwives seemed ready to provide support to newly qualified midwives, even though the support was not based on any formal or structured programme. Lack of commitment to provide mentoring to newly qualified midwives led to frustration and anxiety which negatively affected graduates' confidence. This is consistent with what was reported in a study on ‘clinical practice of midwifery graduates during community service placement, in Limpopo province of South Africa’, SimaneNetshisaulu and Maputle revealed that, graduates felt anxious and frustrated during transition period as they were expected to function all by themselves without mentors or any formal and structured support18. Participants expressed lack of confidence to stand on their own, resulting in shock frustration and anger^18^.

The findings also revealed that newly qualified midwives failed to experience sense of belonging as they felt abandoned and ignored during the time they needed support most. This is supported by what was reported by Cummins et al, whereby graduates to whom a mentor was allocated, valued mentoring process and reported positive relationship which developed between them and the more experienced midwives which was very helpful in boosting their confidence^4^. Graduates who received support from experienced midwives demonstrated a sense of collective, cooperative and collaborative responsibility regarding provision of support through mentorship; felt they belonged to the team of midwives, which motivated them to work confidently^6^.

Based on the findings of the study, midwifery units were not conducive for clinical support and learning; as there was no formal mentoring process in place. When graduates raised, questions related to care provision, experienced midwives did not attend to them. Some experienced midwives were not supportive. Newly qualified midwives had to learn through trial and error, which put patients' lives at risk. Therefore, graduates were denied the opportunity to learn, which negatively affected their competence. Hence, effective mentoring is necessary for establishment of a supportive professional environment which enhances competency and professionalization, thus resulting in positive patient care outcomes. This was supported by Tarimo, Moyo, Masenga, Magesa and Mzava who asserted that, effective and good quality learning environment should be created as it serves as a form of support for newly qualified midwives during transition period^19^. The same sentiments were echoed by Khunou, when reporting that the mentor has an important role to play in supporting and guiding graduates to enhance professional growth and maturity^20^,^21^. Kensington et al, asserted for a supportive working environment when they advocated for provision of a mentor as well as continued clinical knowledge building and education for midwifery graduates^6^.

According to the findings of the study, experienced midwives recognised their role in mentoring and supporting graduates as they believed that mentoring promotes professional and personal development of the graduates, however, they felt they did not have sufficient knowledge and skills regarding the mentoring process. This is in line with the findings of the study conducted by Mubeezi and Gidman, in which mentors valued role modelling and provision of knowledge as key priorities of support, however, perceived that they had gaps in the knowledge and skills necessary for mentoring^11^. The authors further reported that mentors had confidence in teaching clinical skills but needed more support regarding theoretical background which formed the basis of clinical skills and practice^11^. In the same study, mentors also reported that they needed update sessions instead of relying on their own training as the basis for passing on knowledge to others^11^.

Experienced midwives reported shortage of staff and increased workload as a burden which hindered their mentoring role. Results also revealed shortage of resources as a challenge associated with the role of mentor. This was supported by Mubeezi and Gidman who reported that increased workload due to large numbers of patients and staff shortages resulted in limited time for mentoring^11^. The findings of the study conducted by Simane-Netshisaulu et al. concurred with those of Mubeezi and Gidman, when they reported shortage of staff with increased workload as a factor that led to lack of mentoring of newly graduated midwives during transition period; which negatively affected graduates' performance^11^,^15^,^22^.

The student confirmed the challenges of lack of effective mentoring of graduates in the African continent, which seems to be common as supported bywhatwas reported in Nigeria that newly qualified midwiveswereleft with no choice, but just to practice through trial and error, as there was nobody to rely on for support, supervision and mentoring which put patients' lives at risk^23^. In a study conducted in Swaziland, newly qualified midwives requested that they be supported through mentoring as they felt inadequate as far as provision of midwifery services was concerned^24^.

On the contrary, in Singapore, the National University Hospital which is a member of the National University Health System; developed a 3-year structured program specifically designed to address the identified needs of new graduates. During the first year, emphasis is given to induction and opportunities to develop clinical competence and confidence through a close working relationship with a registered nurse buddy. In Year 2, graduates commence a one-year structured mentorship program which focuses on workplace relationship issues, and provides a dedicated mentor for coaching in professional development and fostering a sense of professional identity. Year 3 focuses on clinical rotations, professional role consolidation, and preparation for an area of specialty practice^25^. The study indicatedthe need to implement similar mentorship programmes in developing countries to support and coach new graduates.

## Conclusion

The findings of the study revealed that, newly qualified midwives were not effectively mentored during transition period. Only a few experienced midwives seemed ready to provide support to newly qualified midwives, though the mentoring they provided was informal and unstructured. Experienced midwives recognised their mentoring role however, felt they did not have sufficient knowledge and skills regarding mentoring process. Shortage of staff and increased workload were reported burdens which negatively affected experienced midwives' role as mentors. Formal and structured mentoring programme for supporting newly qualified midwives should be put in place, otherwise competence and confidence level of graduates are compromised. This in turn negatively affects the quality of care provision to patients.

## Recommendations

The following are the recommendations made by the researchers:

Experienced midwives should be trained to empower them with the necessary knowledge and skills regarding mentoring process. Refresher courses should be implemented to keep experienced midwives up to date with new developments, so that they can be able to address challenges experienced by graduates during care provision. The number of nurses and midwives should be increased to enable them to managethe workload, therefore they will have enough time to attend to the support needs of newly qualified midwives.

## Limitation

Thatnursing service managers were supposed to have formed part of the participants as they are responsible for staff establishment, development and training of mentors and provision of equipment necessary for enhancing mentoring process. Future research should be conducted focusing on development of a mentoring programme to facilitate effective transition process for newly qualified midwives.

## Authors' Information

Khathutshelo Grace Simane-Netshisaulu: PhD, Lecturer, Department of Advanced Nursing Science, University of Venda, Limpopo Province, South Africa. Maria Sonto Maputle: PhD, Professor, Department of Advanced Nursing Science, University of Venda, Limpopo Province, South Africa. Mutshinyalo Lizzy Netshikweta: PhD, Professor, Department of Advanced Nursing Science, University of Venda, Limpopo Province, South Africa. Hilda Shilubane: PhD, Professor, Department of Advanced Nursing Science, University of Venda, Limpopo Province, South Africa.

## Abbreviations

RCM, Royal College of Midwives; RSA, Republic of South Africa; SANC, South African National Council; SANDoH, South African National Department of Health; UNMC, Ugandan Nurses and Midwives Council.

## Data Sharing Statement

The data will be available from the corresponding author upon request.
